# An impact of economic slowdown on health. New evidence from 21 European countries

**DOI:** 10.1186/s12889-022-13740-6

**Published:** 2022-07-23

**Authors:** Paweł Prędkiewicz, Agnieszka Bem, Rafał Siedlecki, Milena Kowalska, Marlena Robakowska

**Affiliations:** 1 Wrocław University of Economics and Business, Wrocław, Poland; 2 Medical University of Gdańsk, Gdańsk, Poland

**Keywords:** Economic crises, Health, Unemployment, GDP, Public finance

## Abstract

**Background:**

The economic slowdown affects the population's health. Based on a social gradient concept, we usually assume that this detrimental impact results from a lower social status, joblessness, or other related factors. Although many researchers dealt with the relationship between economy and health, the findings are still inconsistent, primarily related to unemployment. This study reinvestigates a relationship between the economy's condition and health by decomposing it into macroeconomic indicators.

**Methods:**

We use data for 21 European countries to estimate the panel models, covering the years 1995–2019. Dependent variables describe population health (objective measures – life expectancy for a newborn and 65 years old, healthy life expectancy, separately for male and female). The explanatory variables primarily represent GDP and other variables describing the public finance and health sectors.

**Results:**

(1) the level of economic activity affects the population’s health – GDP stimulates the life expectancies positively; this finding is strongly statistically significant; (2) the unemployment rate also positively affects health; hence, increasing the unemployment rate is linked to better health – this effect is relatively short-term.

**Conclusions:**

Social benefits or budgetary imbalance may play a protective role during an economic downturn.

**Supplementary Information:**

The online version contains supplementary material available at 10.1186/s12889-022-13740-6.

## Background

The economy affects health. At the same time, health impacts, at least partially, the economy. Although this is a two-way relationship, researchers usually assume that the economy affects health more significantly than vice versa. The social gradient shapes health and causes inequalities [1, 2].

As a world and individual country, we witness the next economic crisis due to consecutive waves of the pandemic. Even though this economic depression is rooted in the COVID-19 epidemic, its social and economic implications would be a slower pace of economic development, decreasing incomes, and higher unemployment rates. Many countries have already reported a wave of bankruptcies and thousands of new jobless.

Economic lockdown is amplified by the need for social isolation imposed by most countries [3–5]. This situation will require various actions, including monetary, fiscal, and health policy responses [6]. It inspired us to reinvestigate the relationship between the economic crisis and the population's health, bearing in mind that it was not directly COVID-19 that triggered the economic slowdown but the measures taken to limit its spreading.

It is essential that, although studies in this area are relatively numerous, the results are questionable and inconsistent—with this study, we try to be a part of this academic discussion. We investigate the consequences of economic depression based on the experience of the last economic crisis that struck European countries at the end of 2008 and caused a substantial public finance crisis. Consequences spread across European public sectors affecting the labour market, household income, education, health, and social policy. The last financial crisis manifested itself in many ways: the significant decrease in GDP, the rapid growth of unemployment, and, finally, the increase in public debt [7, 8].

When it comes to health systems, they were under pressure to rapidly adapt to market conditions when public health financing decreased due to implemented austerity measures. The governments react in this situation twofold – making budgetary cuts and providing essential services targeted at the poorest [9], or implementing an active policy preventing the wave of bankruptcies, maintaining consumption, and stimulating economic growth, even if scarifying a budgetary balance.

The health budgets reacted to these new economic conditions dissimilarly. Some countries implemented significant restrictions [10–13], which, according to Bosch and colleagues, was not the best strategy [14]. In some states, the funds were frozen, usually since there were earmarked for health care (as in compulsory health insurance). Other studies show that budgets were not restricted, but their growth was notably slower [15, 16], affecting changing demographic structure. Studies also suggest that countries with compulsory health insurance models, where the funds depend on remuneration, suffered a more important worsening of health status than countries with Beveridge’s schemes [17], where funds are usually less flexible and budgeted in advance. They were also more likely to implement the targeted cuts [18]. Some countries leapt at the opportunity to cut costs, especially in hospitals [19] and the pharmaceutical industry [13, 20]. Others lowered or froze the salaries of healthcare professionals [7, 13].

A lower level of public financing also forced cost-sharing—generally in the form of higher deductibles or co-payment [7], [10–12], [21–25]. It changed the structure of healthcare financing by increasing the percentage of private funds [12, 26] and the situation of most vulnerable groups [23], especially the elderly [24, 27].

]The crisis hits not only public budgets but also affects individuals and households. Several studies confirm the link between socioeconomic determinants and health [28], suggesting that income is one of the most critical factors [1], [29–32], but in terms of the economic crisis, other factors also acquire significance [28], especially low income and job insecurity [21], [28], [33–35]. The labour market conditions force workers to be more active [36], and extended working hours may harm the population’s health collaterally [37].

Reduced funding may force healthcare providers, depending on the system’s incentives, to limit the number of consultations or inpatient treatment, and extend the waiting times in a quest for cost-cutting [7, 21]. It potentially reduces access to medical treatment [7] and increases the level of unmet health needs. Kentikelenis and colleagues report a lowering number of GP consultations and, at the same time, a higher ratio of admissions to public hospitals [19]. Also, Perelman and colleagues observe the higher rate of hospital discharges and urgent stays. We observe this pattern when access to primary care is limited. It results from lower funds for preventive services and health education [37].

All the above factors, combined or independently, affect the population’s state of health expressed by indicators of overall mortality or life expectancies [11, 33, 34, 38]. There are several potential consequences of the economy’s collapse: increased risk of cardiovascular disease [33], increasing incidence of infectious diseases [11], or just lower self-perceived health [8, 21, 33]. On the other hand, previous research must not fully confirm these outcomes – Granados and Rodriguez find that the population’s health improved during the last economic crisis [39]. Also, Regidor and colleagues report lowering all-cause mortality [40], just as Laliotis and colleagues [41]. Those observations are supported by Ruhm’s findings, which show that mortality becomes unrelated to macroeconomic conditions [42].

## Methods

We analyse data for 21 European countries (EU countries): Austria, Belgium, Czechia, Denmark, Finland, France, Germany, Greece, Hungary, Ireland, Italy, Latvia, Lithuania, Netherlands, Poland, Portugal, Slovakia, Slovenia, Spain, Sweden, United Kingdom. Based on the literature review, we formulate the following research questions:

1. What is the relationship between the economy’s condition and health? Which indicators of the macroeconomic situation affect health more profoundly?
2. What is the strength of this relationship? Do they stimulate health positively or negatively?
3. Are there differences between men's and women's subpopulations?

We verify the above questions by estimating balanced panel data models. Table 1 presents the list of variables considered in this study; it is split into three categories:

- health state indicators,
- health system indicators representing financial and human resources,
- macroeconomic indicators, describing the economy’s condition.

**Table 1:** Analysed variables

Variable	Description	Source	Availability of data
*Health state*			
Life Expectancy (LE0, LE65)	The mean number of years a newborn child (LE0)/a person at the age of 65 (LE65) can expect to live if subjected to the current mortality conditions, the probabilities of dying at each age	Global Burden of Disease (GBD)	1990–2019
Healthy life expectancy, or Health-adjusted life expectancy (HALE0, HALE65)	The number of years that a person at a given age (a newborn – LE0 and a person at the age of 65 – LE65) can expect to live in good health if the rates of all-cause mortality and all-cause disability in a specified year of interest would remain constant into the future	Institute for Health Metrics and Evaluation	1995–2019
Infant Mortality (IM)	The number of deaths under one year of age	World Bank	1990–2019
Suicide rate (SUICIDE)	Suicide mortality rate is the number of suicide deaths yearly — crude suicide rate (not age-adjusted)	OECD	1961–2019
*Health system*			
Current Health Expenditure (CHE)	Level of current health expenditure include consumed healthcare goods and services	World Bank	2000–2019
Total Health Expenditure (THE)	Health expenditure includes all expenditures for the provision of health services, family planning activities, nutrition activities and emergency aid designated for health, but it excludes the provision of drinking water and sanitation	WHO Health for All	1995–2019
Hospital Beds (BEDS)	Total hospital beds include curative care beds, rehabilitative care beds, long-term care beds, and other beds in hospitals	Eurostat (Health)	1990–2015
*Macroeconomics*			
Gross Domestic Product (GDP)	The measure of the value-added created through the production of goods and services in a country during a specific period. GDP PPP per capita	European Health for All database WHO	1990–2018
Total investment (TOT_INV)	Investment or gross capital formation measured by the total value of the gross fixed capital formation and changes in inventories and acquisitions less disposals of valuables for a unit or sector. Expressed as a ratio of total investment to GDP	International Monetary Fund (IMF)	1990–2019
Unemployment rate (UNEMP)	The number of unemployed people as a percentage of the labour force, where the latter consists of the unemployed plus those in paid or self-employment	World Bank	1991–2019
General Government Balance (GOV_BAL)	Defined as the balance of income and expenditure of government, including capital income and capital expenditures	Euro-stat	1995–2018
SOC_BEN	Social benefits to households covering social benefits other than social transfers in kind; and social transfers in kind	OECD	1995–2015

Data come from WHO, World Bank, IMF, OECD, GBD and Eurostat databases. The availability of individual variables strongly determines the selection of the dataset. We exclude several candidate variables due to short time series or missing data points. These are, for example, unmet health needs, number of doctors/nurses per 1.000 inhabitants, investment, Gini coefficient, the share of the population at risk of poverty.

Due to data availability, we limit our research time to 1995 – 2019, building a fixed and balanced panel.^1^ We estimate the heteroskedasticity and autocorrelation consistent models (robust HAC standard errors) based on Newey–West estimator. The models take the following form:

$${Y}_{t,i}={a}_{0} +{a}_{1}*{SOC\_BEN}_{i,t}+{a}_{2}*{TOT\_INV}_{i,t}+{a}_{3}*{UNEMP}_{i,t}+{a}_{4}*{GOV\_BAL}_{i,t}+{a}_{5}*{CHE}_{i,t}+{a}_{6}*{BEDS}_{i,t}+{a}_{6}*{LO{G\_GDP\_PPP}_{i,t}}+{a}_{7}*{Y}_{i,t-1}+{COUNTRY\_FE}_{i}+{\varepsilon }_{i,t}$$

where: i—countries.

We employ a backward stepwise regression procedure by gradually eliminating statistically insignificant variables from the regression model to find a reduced model that best explains the data [43, 44].

Strong autocorrelation in the analysed time series is a source of the most critical estimation problems. First, we employ the Newey–West estimator, but it occurs to be insufficient (Durbin-Watson test statistics in the range 0.15–0.6). Hence, we are forced to add lagged dependent variable Y_t-1_ [45, 46], being aware that it may suppress an explanatory power (potential underestimation) of independent variables [47].

This panel analysis also allows controlling for unobserved or omitted time-invariant individual effects by including country dummy variables, which may otherwise bias the estimation (culture, diet). At the same time, to improve the model's strength, we abandoned the use of time dummies.

The statistic tests are employed to choose between fixed and random effect models. First, we use the Breusch-Pagan test to control heteroscedasticity. The null hypothesis assumes that the variance of the unit-specific errors is equal to 0 (homoscedasticity). Accepting the null hypothesis (p-value higher than the threshold α) supports the homoscedasticity hypothesis—it excludes the model with random effects (RE).

In the next step, we employ the Hausman test for the models where both random effects (RE) and fixed effects (FE) are potentially consistent. The null hypothesis is that the preferred model is random effects. The alternate hypothesis is that the model with fixed effects (FE) is consistent. Based on the p-value, we choose the models that better fit the data. The rest results are presented in Table 2. For all dependent variables, FE models are consistent. We use Gretl software to support the calculations.

**Table 2:** Dependent variables

Variable	Abbreviation	Unit
Life expectancy for a female newborn	LE0_F	Years
Life expectancy for a female aged 65	LE65_F	Years
Life expectancy for a male newborn	LE0_M	Years
Life expectancy for a male aged 65	LE65_M	Years
Life expectancy in good health for a female newborn	LE0_F	Years
Life expectancy in good health for a female aged 65	LE65_F	Years
Life expectancy in good health for a male newborn	LE0_M	Years
Life expectancy in good health for a female newborn	LE0_F	Years

The problem of potential multicollinearity in the dataset is controlled using Variable Inflation Factors (VIF), which determines the strength of the correlation between the independent variables. It is predicted by taking a variable and regressing it against every other variable. We assume that the maximum acceptable level of VIF is 10.

### Dependent variables

The effectiveness of health systems can be described by the production of services (health outputs—number of procedures, person-days) or health outcomes (life expectancies, morbidity, or mortality) [48–53]. Apart from those “objective” measures, some studies refer to subjective measures like health self-assessment or subjective well-being [54].

This research concentrates on objective measures describing health outcomes that better compare countries with different cultures affecting subjective measures (like self-perceived health state). We reject the disease-related models at the planning stage, potentially biased by differences in reporting morbidity or, purely, diagnostic procedures availability. Since sex is a factor that significantly differentiates healthy life expectancy, we estimate some models separately for the male and female populations (Table 2).

Both life expectancies and mortality are confirmed in the literature and accepted by WHO as objective indicators of health systems’ performance. Life expectancies are based on a synthetic cohort and are therefore not the LE of anyone. Hence it cannot assess the relative risk but describes the public health system as a synthetic measure of mortality [55]. HALE also measures the health systems’ performance by incorporating premature mortality and non-fatal health outcomes in a population. It shows the expected life expectancy in good health [56].

### Explanatory variables

As we analyse the economic crisis’s influence on the health systems’ performance, the explanatory variables represent the economy’s activity indicators. In the case of GDP, as a dominant measure of the economy’s activity, we employ a logarithm for two reasons: to reduce the differences in scale between countries and be in line with the Preston law [57] (Table 3).

**Table 3:** Explanatory variables

Variable	Abbreviation	Unit
Gross Domestic Product	LOG_GDP	The logarithm of GDP per capita, PPP (constant 2017 international $)
Social benefits	SOC_BEN	% of GDP
Total Investment	TOT_INV	% of GDP
Unemployment	UNEMP	% of the labour force
General Government Balance	GOV_BAL	% of GDP
Current Health Expenditure	CHE	% of GDP
Hospital beds	BEDS	The number per 100.000 population

To extend the analysis, we introduce two measures of health system resources (infrastructural and financial – CHE, BEDS), which describe the size and distribution of funds [45, 58]. We base on the assumption that although health spending (expressed in %GDP) shows some volatility, which, in the context of previous studies, might be evident during the economic crisis, the infrastructure resources represent significant inertia in response to macroeconomic or demographic changes. The system’s size cannot be significantly limited in a short period, even if the governments implement restrictions. As the infrastructural resources can generate the specified health outcomes, we interpret them as the variables to control the health system’s effects.

## Results

### *Models without lags*^2^

Table 4 presents the Breusch-Pagan test and the Hausman test for estimated RE models. We do not diagnose homoscedasticity in all models so that we can employ both RE and FE estimation. Hence, we fail to accept the null hypothesis of homoscedasticity for all models. Simultaneously, the Hausman test shows that the FE is preferred for all estimated models.

**Table 4:** Fixed Effects vs Random Effects—tests results

	Breusch-Pagan test (Null hypothesis: Variance of the unit-specific error = 0)	Durbin-Wu-Hausman test (Null hypothesis: GLS estimates are consistent)
	Asymptotic test statistic: Chi-square	Asymptotic test statistic: Chi-square
LE0_M (model 1)	2391.06 ^***^	33.0661 ^***^
LE65_M (model 2)	1120.84 ^***^	70.9523 ^***^
HALE0_M (model 3)	2768.23 ^***^	30.3985 ^***^
HALE65_M (model 4)	2406.02 ^***^	9.76034 ^*^
LE0_F (model 5)	2391.06 ^***^	33.0661 ^***^
LE65_F (model 6)	2651.24 ^***^	28.0398 ^***^
HALE0_F (model 3)	2615.73 ^***^	19.3224 ^***^
HALE65_F (model 4)	2549.78 ^***^	56.1893 ^***^

^*^ significance level α = 0.1

^**^ significance level α = 0.05

^***^ significance level α = 0.01

Table 5 presents four estimated models for the male populations. The estimated models suggest that a level of total investments affects negatively all dependent variables describing the health status of the male populations. The strength of those relationships is higher for life expectancies for a newborn. There are statistically significant at the level of *p*-value < 0.05. Hence, a lower level of investments contributes to the longer life expectancies for the male population.

**Table 5:** Panel data models with FE for the male population (models 1–4)

Y	LE0_M (model 1)	LE65_M (model 2)	HALE0_M (model 3)	HALE65_M (model 4)
Y_1	0.88049 (0.02027) ^***^	0.89589 (0.009) ^***^	0.88484 (0.02154) ^***^	0.89942 (0.01014) ^***^
const	-3.47512 (1.5864) ^**^	-3.36433 (0.60715) ^***^	-2.37814 (1.38767)	-2.19801 (0.42005) ^***^
SOC_BEN				0.00884 (0.00299) ^***^
TOT_INV	-0.0188 (0.00757) ^**^	-0.00755 (0.00309) ^**^	-0.01469 (0.0059) ^**^	-0.00532 (0.00203) ^**^
UNEMP	0.01757 (0.00555) ^***^	0.00567 (0.00274) ^*^	0.01525 (0.00472) ^***^	
GOV_BAL	-0.01067 (0.00396) ^**^	-0.00597 (0.00123) ^***^	-0.00943 (0.00329) ^***^	-0.00415 (0.00089) ^***^
LOG_GDP_PPP	2.84368 (0.67378) ^***^	1.16824 (0.15165) ^***^	2.27663 (0.60561) ^***^	0.77324 (0.11564) ^***^
RHO	0.062	-0.059	0.097	-0.027
LSDV-Rsq	0.9819	0.985	0.9833	0.9856
No._of_observation	420	420	420	420
Max VIF	5.21	4.36	5.37	3.61

^*^ significance level α = 0.1

^**^ significance level α = 0.05

^***^ significance level α = 0.01

Analogous relationships exist between a budgetary balance and life expectancies. The direction of dependence is negative, which means that striving to maintain budget balance (reducing the deficit) harms the male population's health. This relationship is highly statistically significant (*p*-value < 0.01, except for the life expectancy for male newborns, where the p-value is lower than 0.1).

In the GDP case, we observe a positive relationship—a higher level of economic activity extends the life expectancies of men. This effect is more substantial in the case of newborns. This relationship is highly statistically significant (in all models, *p*-value < 0.01).

As for the impact of unemployment on health, the estimated models indicate a positive relationship between these phenomena. The increase in unemployment thus extends the life expectancies. This relationship is weaker for men aged 65 years (statistical significance at *p*-value < 0.1 for the life expectancy for men aged 65, and statistically insignificant for healthy life expectancy for men aged 65).

In the case of healthy life expectancy for men aged 65, we also observe a positive relationship between social security spending and the healthy life expectancy for men aged 65 (*p*-value < 0.01).

For women, we observe a similar pattern of dependencies (Table 6). As with men, there is a strong and statistically significant (*p*-value < 0.01) relationship between GDP and women's life expectancies. We also identify a negative relationship between budget balance and life expectancy, as in the case of men. This relationship is statistically significant for most dependant variables (*p*-value < 0.01, except for life expectancy for women aged 65, where *p*-value < 0.1). Women's health is also affected by the situation in the labour market. As in the case of men, the increase in unemployment contributes to the longer life expectancy – both for life expectancy for newborns and women aged 65 (*p*-value < 0.01 or 0.05).

**Table 6:** Panel data models with FE for the female population (models 5–8)

Y	LE0_F (model 5)	LE65_F (model 6)	HALE0_F (model 7)	HALE65_F (model 8)
Y_1	0.88189 (0.01509) ^***^	0.88466 (0.00733) ^***^	0.88189 (0.01749) ^***^	0.89195 (0.01174) ^***^
const	2.73211 (0.60243) ^***^	-1.88435 (0.64631) ^***^	2.90169 (0.5532) ^***^	-2.14832 (0.53304) ^***^
UNEMP	0.01256 (0.0035) ^***^	0.01026 (0.00249) ^***^	0.0099 (0.00356) ^***^	0.00527 (0.0022) ^***^
GOV_BAL	-0.00815 (0.00282) ^***^	-0.00454 (0.00218) ^*^	-0.00722 (0.00244) ^***^	-0.00436 (0.0013) ^***^
CHE		0.01463 (0.00819) ^*^		
BEDS				0.0002 (0.0001) ^*^
LOG_GDP_PPP	1.56704 (0.27941) ^***^	0.89761 (0.14669) ^***^	1.20373 (0.29808) ^***^	0.76851 (0.12963) ^***^
RHO	-0.21	-0.14	-0.15	-0.13
LSDV-Rsq	0.9817	0.9822	0.9822	0.9827
No._of_observation	420	420	420	420
Max VIF	3.52	3.66	2.39	3.18

^*^ significance level α = 0.1

^**^ significance level α = 0.05

^***^ significance level α = 0.01

Models 6 and 8 also identify relationships that do not appear in models estimated for men. Older women's health is also positively influenced by factors related to a health care system. First of all, the amount of current expenditure on health care positively stimulates the life expectancy of women aged 65. For women aged 65, the availability of hospital beds also plays a vital role in extending healthy life expectancy. Those dependencies are positive and statistically significant (*p*-value < 0.1). Other variables, like the level of investments or social spending, are statistically insignificant.

### Models with lags^3^

The models described above identify the dependencies between variables in the same period. However, intuition suggests that there may be some delays between explanatory and dependent variables. Thus, unemployment or GDP from previous periods may explain health in a given year. We verify this assumption by analysing the correlations between variables. The results suggest a substantial lag for the following variables: unemployment, current health spending, number of hospital beds, total investments and GDP. We analyse the lags from 1 to 5 years. We select the explanatory variables based on a correlation matrix according to the following rule: we analyse the most correlated variable (with lags or without lags) for every variable (Table 7).

**Table 7:** Lags in explanatory variables in models 8–16

Variable	Lag (years)
LOG_GDP	5
SOC_BEN	0
TOT_INV	3
UNEMP	5
GOV_BAL	0
CHE	0
BEDS	5

Considering the potential lags, we estimate additional models 9–16, separately for men (Table 8) and women (Table 9). Models with delayed variables show that only two variables (GDP with lag [t-5] and the number of hospital beds with lag [t-5]) affect men's health with a more significant delay. First of all, there is a highly statistically significant (*p*-value < 0.01) relationship between the level of economic activity – the strength of this relationship is almost the same as in models without delays.

**Table 8:** Models 9–12, male population, explanatory variables with lags

Y	LE0_M (model 9)	LE65_M (model 10)	HALE0_M (model 11)	HALE65_M (model 12)
Y_1	0.85519 (0.0134) ^***^	0.898 (0.00811) ^***^	0.8587 (0.01623) ^***^	0.90229 (0.00857) ^***^
const	1.13909 (1.16633)	-2.32126 (0.36463) ^***^	1.32666 (1.07139)	-1.78237 (0.2761) ^***^
SOC_BEN	0.02806 (0.01357) ^*^		0.02538 (0.01036) ^**^	
GOV_BAL		-0.0043 (0.00115) ^***^		-0.00341 (0.00076) ^***^
BEDS_5	-0.00061 (0.00016) ^***^		-0.00046 (0.00016) ^**^	
LOG_GDP_PPP_5	2.208 (0.30453) ^***^	0.91552 (0.09521) ^***^	1.80267 (0.27635) ^***^	0.68337 (0.07234) ^***^
RHO	0.042	-0.062	0.074	-0.035
LSDV-Rsq	0.9824	0.9847	0.9838	0.9854
No._of_observation	420	420	420	420
Max VIF	8.16	3.34	7.59	3.25

^*^ significance level α = 0.1

^**^ significance level α = 0.05

^***^ significance level α = 0.01

**Table 9:** Models 13–16, female population, explanatory variables with lags

Y	LE0_F	LE65_F	HALE0_F	HALE65_F
Y_1	0.86316 (0.01398) ^***^	0.8863 (0.01063) ^***^	0.85939 (0.01808) ^***^	0.87982 (0.01299) ^***^
const	5.72624 (0.74034) ^***^	-0.69169 (0.2296) ^***^	5.15643 (0.79455) ^***^	-0.58295 (0.15798) ^***^
SOC_BEN	0.01927 (0.00835) ^**^	0.00749 (0.00392) ^*^	0.01798 (0.00678) ^**^	0.00735 (0.00368) ^*^
GOV_BAL		-0.00411 (0.00197) ^**^		-0.00281 (0.00147) ^*^
BEDS_5	-0.00025 (0.00006) ^***^		-0.00014 (0.00007) *	
LOG_GDP_PPP_5	1.21587 (0.13269) ^***^	0.65883 (0.07354) ^***^	1.01342 (0.15192) ^***^	0.5158 (0.06149) ^***^
RHO	-0.22	-0.16	-0.12	-0.12
LSDV-Rsq	0.9818	0.9823	0.9824	0.9826
No._of_observation	420	420	420	420
Max VIF	4.10	2.74	2.48	2.46

^*^ significance level α = 0.1

^**^ significance level α = 0.05

^***^ significance level α = 0.01

When we consider delays, men's health is also negatively affected by the number of hospital beds (with lag [t-5]) – this relationship, statistically significant (*p*-value 0.01 or 0.05), occurs for life expectancy and healthy life expectancy for males newborns.

This estimation also identifies the relationships between men's health and social spending and health and budget balance. Similarly, as in the case of models without delays, maintaining a budget balance harms health, but we see it only in the case of life expectancy and healthy life expectancy for male newborns, although it is a very statistically significant relationship (*p*-value < 0.01).

As far as social spending is concerned, current expenditure on social benefits positively impacts life expectancy (and healthy life expectancy) for male newborns; however, this relationship is less statistically significant (*p*-value < 0.05 or 0.1).

Similarly to men, women's health is positively stimulated by the level of economic activity (with lag [t-5]). This dependency is highly statistically significant (*p*-value < 0.01). We also observe a negative relationship between the number of hospital beds and health, also present in models for the male population. This relationship occurs only for life expectancy and healthy life expectancy for female newborns, but only for overall life expectancy. It is strongly statistically significant (*p*-value < 0.01).

Estimated models also confirm the negative impact of governmental balance on health, but the significance of those dependencies (identified only for life expectancy and healthy life expectancy for females aged 65) is lesser than for men. However, the general pattern of dependence is the same as that of men.

Models with lags also show that social spending can positively impact women's health (for all variables explained). This relationship is more statistically significant for life expectancy and healthy life expectancy for female newborns. Interestingly, models with delayed variables do not identify unemployment as an essential factor contributing to longer life expectancies.

To summarise our findings:

1. The level of economic activity significantly affects the population’s health—both men and women. Higher GDP positively stimulates life expectancies; this finding is strongly statistically significant. We can observe this impact both in models with lags or based on current values of variables;
2. We also observe a negative impact of a higher volume of investment. This effect is statistically moderately significant and occurs primarily in the case of men. It seems surprising since investments generally have a positive impact on economic development. However, financing investments, especially from internal funds, leads to a reduction in current expenditure. It may be the mechanism lying behind the identified negative impact;
3. The impact of the social benefits and the budgetary balance should be discussed together. First, estimated models suggest that social benefits, including benefits related to unemployment, do not seem to be an essential factor explaining the impact of the economy on health. The positive impact of social benefits on health appears only in models with delayed variables, especially for women.At the same time, we observe that the pursuit of budgetary balance harms health. Budgetary balance, especially in times of crisis, is usually achieved by reducing budgetary spending, often of a social nature. This negative relationship appeared in almost all estimated models (with and without delays)^4^;
4. We also confirm that the unemployment rate positively affects health. Hence, the increasing unemployment rate contributes to better health. This effect is highly statistically significant for both women and men. However, we only identify such relationship in models without delay, suggesting that there is no a long-term, deferred impact of unemployment on a health state when expressed using the positive measures;
5. Additionally, we confirm the negative relationship between the number of hospital beds and health. It can be interpreted in the context of healthcare spending structure – when more funds are consumed by stationary healthcare, a healthcare system is less effective. It can be reflected in the life expectancies.

## Discussion

The results support the belief that the economic crises, which reveals in GDP reduction, negatively affect health [11, 33, 34, 38, 46]. Cavicchioli and Pistoresi estimate that an increase of 1% in real GDP reduces mortality by 0.27% [59]. When the GDP decreases or even its growth rate decreases, it adversely affects the population’s health. This finding is not surprising as income, or broader speaking—wealth, is one of the critical social determinants of health, although several studies collide with this observation, among all the works of Gerdtham and colleagues [60–62]. This finding has one significant limitation – due to the data structure, we cannot identify potential health disparities between different socioeconomic groups that may dissimilarly react to changes in the economic situation.

These are also essential issues which our research does not confirm. As our results suggest, this impact is not transmitted by increasing health spending. It is in line with Cima, who concludes that expenditure growth is a less productive path to improve a population’s health than increasing GDP [17]. Moreover, developing the health system's size by increasing the number of hospital beds may not be the best strategy for population health [63, 64].

The study also confirms that restrictive budgetary policy may harm the population’s health, especially during economic crises. Cutting public spending may cause health deprivation, especially for vulnerable groups. Public spending, particularly for social purposes, can play a protective role during periods of crisis. This finding is in line with some previous studies [65–69].

According to the unemployment problem, all estimated models indicate a positive impact of unemployment on the population’s health (higher unemployment rates are linked to longer life expectancies or overall health). This conclusion is at odds with many previous studies situating unemployment as one of the leading health determinants and highlighting its adverse impact [21, 28, 33, 34, 37, 70]. At the same time, our findings are in line with some newest studies [38, 42, 61], [71–74]. Although correlation analysis indicates some lags in this relationship, the models do not confirm that unemployment from earlier periods has a more substantial impact on health than the current situation.

This effect requires further research. Hence, it can be interpreted from two perspectives. First, all analysed countries offer medical services for the unemployed, although the range of services is usually limited. Of course, as a typical result of a loss of work, a reduction in income can lower access to benefits if it involves a fee. However, in systems based on public healthcare, the waiting lists are the primary mechanism for rationalising medical services access. Therefore, the lack of work-related obligations may increase the possibility of obtaining medical benefits. As a second thing, unemployment can be a source of stress, but those who work are sometimes under more pressure, negatively affecting their health [75]. Losing a job also enables taking personal care of family members (e.g. parents), positively impacting the overall population’s health.

## Conclusions

To conclude, we confirm the relationship between the economy’s condition and health – it is transmitted via lowered GDP and higher unemployment rates. At the end of the day, the level of generated GDP, not unemployment, is a crucial factor that shapes the population’s health in a crisis (GDP and unemployment are correlated at 25–30%).

The crisis hits not only by the slower pace of economic growth but also by higher unemployment rates. According to the literature, unemployment, particularly long-term, affects health status by reducing income, losing status related to professional position and reducing social interaction. However, in light of our research, it does not adversely affect the population's health. We cannot also confirm the long-term impact of unemployment on health. We consider this finding particularly relevant in the context of previous inconsistent results. We can also confirm that other factors, like social benefits or budgetary imbalance (via extended budgetary spending), may smooth the detrimental impact of the economic crisis.

According to the relationship between the economy and health, the source of inconsistency may also be rooted in research methods. Our first observations, but not supported by broader analysis, suggest that a dependent variable’s choice may affect the results. When a dependent variable is expressed as an objective measure like mortality or life expectancy, newer studies usually detect a countercyclical relationship between economic situation and health. At the same time, research suggests a procyclical association when the dependent variable is subjective, like health state self-assessment.

The debate on the relationships between the economy and health is still open. We hope to contribute to this academic discussion by providing new findings. The turn of the twentieth and twenty-first centuries shows how sensitive is the global economy to economic perturbations, and the COVID-19 pandemic has made it clear that the sources of the subsequent crises may lie far from the financial markets. That makes this subject topical.

## Supplementary Information

**Additional file 1.** 

## Data Availability

The datasets used and/or analysed during the current study are available from the corresponding author on reasonable request.

## Abbreviations

BEDS: Hospital Beds per 100,000 inhabitants
CHE: Current Health Expenditure measured in % of GDP
HALE: Health-adjusted life expectancy
GDP PPP: Gross Domestic Product Purchasing Power Parity
GOV_BAL: General Government Balance
LE: Life expectancy
SOC_BEN: Social benefits
TOT_INV: Total investments
UNEMP: Unemployment rate

## Footnotes

1 We use, in all models, the variable current health expenditures (CHE). As the international databases cover this variable starting from 2000, the missing observations for the years 1995–1999 were estimated based on the ratio CHE/THE in the year 2000 and THE. Finally, the level of CHE for 1995–1999 is a percentage of THE estimated separately for each country.

2 Models without lags are estimated for the timespan 2000–2019 to keep the results comparable.

3 Estimation based on the timespan t = 2000–2019.

4 When we exclude from the analysis the variable GOV_BAL it reveals a positive effect of SOC_BEN on health.
